# Internet-delivered guided self-help acceptance and commitment therapy for family carers of people with dementia (iACT4CARERS): a feasibility study

**DOI:** 10.1080/13607863.2021.1985966

**Published:** 2021-10-07

**Authors:** Naoko Kishita, Rebecca L. Gould, Morag Farquhar, Milena Contreras, Elien Van Hout, Andrés Losada, Isabel Cabrera, Michael Hornberger, Erica Richmond, Lance M. McCracken

**Affiliations:** a School of Health Sciences, University of East Anglia, Norwich, UK; b Division of Psychiatry, University College London, London, UK; c Department of Psychology, Universidad Rey Juan Carlos, Madrid, Spain; d Department of Biological and Health Psychology, Universidad Autónoma de Madrid, Madrid, Spain; e Norwich Medical School, University of East Anglia, Norwich, UK; f Norfolk and Suffolk NHS Foundation Trust, Older People’s Community Team, Norwich, UK; g Department of Psychology, Uppsala University, Uppsala, Sweden

**Keywords:** caregivers, web-based, online, eHealth, CBT, mindfulness

## Abstract

**Objectives:**

The feasibility of research into internet-delivered guided self-help Acceptance and Commitment Therapy (ACT) for family carers of people with dementia is not known. This study assessed this in an uncontrolled feasibility study.

**Method:**

Family carers of people with dementia with mild to moderate anxiety or depression were recruited from primary and secondary healthcare services in the UK. Participants were offered eight, guided, self-help online ACT sessions adapted for the needs of family carers of people with dementia with optional online peer support groups. Pre-defined primary indicators of success included recruitment of 30 eligible carers over 6 months and ≥70% completing at least two online sessions.

**Results:**

Thirty-three participants (110% of the target sample) were recruited over 6 months and 30 participants (91%) completed two or more sessions, and thus both indicators of success were met. Further, 70% of participants completed seven or all eight sessions, and 27% of participants were lost to follow-up, but none of the reasons for early withdrawal were related to the intervention.

**Conclusion:**

This study supports the feasibility, including recruitment and treatment completion. A full-scale trial to assess the clinical- and cost-effectiveness of the intervention including its long-term effects is warranted.

## Introduction

In many countries, family members are considered to be an essential workforce in caring for people with dementia. The current annual economic cost of dementia in the UK is estimated at £26.3 billion (Prince et al., 2014), with health and social care costs outweighing those of cancer, coronary heart disease and stroke combined (Luengo-Fernandez et al., 2015). Forty-four percent of this annual economic cost of dementia is contributed by unpaid informal carers such as family members (Prince et al., 2014).

Previous systematic reviews demonstrate that the pooled prevalence of depression and anxiety in family carers of people with dementia are 31.2% and 32.1%, respectively (Collins & Kishita, 2020; Kaddour & Kishita, 2020). This prevalence estimate of depression is higher than the pooled prevalence of depression among outpatients diagnosed with a medical condition (Wang et al., 2017). The prevalence estimate of anxiety is substantially greater than family carers of people with other conditions such as cancer (Friðriksdóttir et al., 2011) and stroke (Loh et al., 2017).

Despite the negative consequences of caregiving, many carers receive no or only minimal support for their own psychological needs due to barriers such as mobility constraints and limited availability of skilled therapists (Alzheimer’s Society, 2014). One way to address the challenges of treatment accessibility in this population, and also scalability, is to design a service that can be delivered remotely, accessed at home, and at the time chosen by the participant. Internet-delivered interventions have a strong potential to overcome these challenges.

A recent meta-analysis concluded that psychotherapeutic interventions or interventions that include psychotherapeutic components demonstrate the largest effects on reduction of depressive symptoms in family carers of people with dementia (Cheng et al., 2020). The manualised psychological therapies for carers of people with dementia, which have been researched so far, are mainly based on cognitive behaviour therapy (CBT) and delivered face-to-face. A recent meta-analysis of in-person or telephone CBT for family carers of people with dementia delivered by trained professionals demonstrated a small overall effect of CBT on depression (*d* = 0.34), while no significant overall effect was observed for anxiety (Hopkinson et al., 2019). A meta-analysis focusing solely on internet- or DVD-delivered self-help CBT for family carers of people with dementia demonstrated that an overall effect of CBT on depression appeared reduced in comparison to in-person or telephone CBT (*d* = 0.27) when a self-help format is used (Scott et al., 2016). The effect on carer anxiety was not reported. These figures suggest a need for improvement, particularly in terms of the effective provision of self-help psychological therapies among this population.

Acceptance and commitment therapy (ACT) is an evidence-based psychological treatment, which aims to facilitate psychological flexibility (Hayes et al., 2013). ACT aims to improve one’s psychological flexibility through three sets of skills: stepping back from restricting thoughts and approaching or allowing painful emotions; focusing on the present, connecting with what is happening in the moment; and clarifying and acting on what is most important to do and building larger patterns of effective values-based action (Hayes et al., 2013).

ACT has a strong evidence base for improving outcomes such as mood and quality of life in various populations including people with depression, anxiety, chronic pain and somatic symptoms (A-Tjak et al., 2015; Gloster et al., 2020; Hann & McCracken, 2014). Recent randomised controlled trials (RCTs) of ACT for family carers of people with dementia also demonstrated that in-person ACT delivered by trained professionals can reduce depression and anxiety in family carers (Losada et al., 2015; Marquez-Gonzalez et al., 2020).

Recent reviews of RCTs of internet-delivered guided or non-guided self-help ACT demonstrated that internet-delivered self-help ACT can reduce depression and anxiety, although the majority of included studies targeted either general or student populations; studies that targeted family carers were not identified (Brown et al., 2016; Thompson et al., 2021). A more recent RCT explored the effectiveness of internet-delivered guided self-help ACT for informal carers aged 60 and over providing care to a spouse or child living with or without disease (Lappalainen et al., 2021). Twenty-six percent of participants were caring for a family member with memory-related problems and the findings demonstrated that internet-delivered self-help ACT guided by trained psychology and gerontology students was effective in reducing depression when compared to usual care, while no effects were found for anxiety.

Current evidence suggests that internet-delivered, guided, self-help ACT may have a considerable potential to treat depression and anxiety in family carers of people with dementia. However, prior to a full-scale RCT, a feasibility study needs to be conducted to test the planned methodology and ensure that internet-delivered guided self-help ACT is acceptable to the targeted population. Therefore, this uncontrolled feasibility study aimed to evaluate whether it is feasible to deliver internet-delivered guided self-help ACT within primary and secondary care services in the UK, particularly in terms of recruitment and treatment completion, and whether the intervention is acceptable to family carers of people with dementia.

## Materials and methods

### Trial design

This was a multi-site, single-arm feasibility study conducted in the UK (ISRCTN trial registration number: 18956412). Three sites, one primary care service (i.e. GP practice) and two secondary care services (i.e. NHS mental health trusts) were involved in participant recruitment and delivery of the intervention. Full ethical approval was obtained from the NHS London-Queen Square Research Ethics Committee (20/LO/0025).

### Participants

Recruitment took place between August 2020 and January 2021 (i.e. over 6 months). Participants were recruited via clinician referral from three participating sites and self-referral from the community, including public advertisement in local newspapers and advertisement on a national recruitment website (Join Dementia Research). The study advertisement was also shared with participants who had previously participated in other ethically approved non-interventional dementia studies at the university and who had consented to be contacted about future studies.

The UK’s NICE guideline for managing anxiety and depression (National Institute for Health and Care Excellence, 2011) recommends the use of a self-help approach for individuals with mild-to-moderate clinical presentations. Thus, participants presenting with mild-to-moderate anxiety or depressive symptoms were recruited. Eligible participants were: (a) aged 18 and over, (b) an unpaid carer for a relative with a clinical diagnosis of dementia, (c) identifying as a primary carer in their family and (d) presenting with mild-to-moderate anxiety or depressive symptoms as indicated by the score of 6–15 on the Generalised Anxiety Disorder-7 (Spitzer et al., 2006) or 6–15 on the Patient Health Questionnaire (Kroenke et al., 2001).

Participants were excluded if they were currently (a) receiving psychological treatment, (b) experiencing current difficulties with a severe and poorly controlled psychiatric disorder (e.g. schizophrenia) or other conditions expected to impair treatment engagement, such as self-reported cognitive impairment or (c) without access to the Internet.

### Interventions

#### Online programme iACT4CARERS

Initially, the development of the online programme was discussed with four public members (family carers). They suggested combining some peer support group sessions with the online programme and allowing carers to access the programme from any device including smartphones for improved accessibility. These suggestions were reflected in the development phase. The structure and content of internet-delivered, guided, self-help ACT, which was adapted for family carers of people with dementia, were then decided based on the consensus among ACT experts in the research team and the written protocol was produced. An administrative staff member with no knowledge of any form of psychotherapy including ACT reviewed the protocol to ensure the terms and descriptions used were clear for the public. The prototype of the online programme was piloted by two public members (family carers) to ensure its relevance and the user-friendliness of the online platform.

The final version of the online programme consisted of eight sessions (see Table 1 of Supplemental Material for the detailed description of each session). Each subsequent session was made available to participants 1 week after the completion of the previous session, and participants were encouraged to complete the next session within the week it was made available. Participants were informed that access to the online programme would cease after 12 weeks.

**Table 1.** Characteristics of participants at screening.

Carer variable (range of the scale)	Mean (SD) or *N* (%) Screened (*N* = 79)^a^	Mean (SD) or *N* (%) Invited to the intervention (*N* = 33)
Mean age (years)	63.59 (10.53)	62.00 (10.17)
Age group (years)		
30–39	1 (1%)	0
40–49	6 (8%)	3 (9%)
50–59	21 (27%)	12 (36%)
60–69	25 (32%)	9 (27%)
70–79	22 (28%)	8 (24%)
80–89	4 (5%)	1 (3%)
Sex		
Female	57 (72%)	29 (88%)
Male	22 (28%)	4 (12%)
Highest level of education		
Primary school	0	0
Secondary school	24 (30%)	11 (33%)
Vocational diploma	26 (33%)	11 (33%)
Undergraduate degree	21 (27%)	9 (27%)
Post-graduate degree	8 (10%)	2 (6%)
Employment status		
Part-time	16 (20%)	6 (18%)
Full-time	14 (18%)	6 (18%)
Unemployed	7 (9%)	4 (12%)
Retired	42 (53%)	17 (52%)
Length of being a carer (in months)	52.66 (40.58)	53.00 (19.48)
Relationship with the care recipient		
Wife	24 (30%)	11 (33%)
Husband	15 (19%)	3 (9%)
Partner	3 (4%)	0
Daughter	29 (37%)	17 (52%)
Son	5 (6%)	1 (3%)
Other	3 (4%)	1 (3%)
Cohabitation status		
Living with the care recipient	50 (63%)	20 (61%)
Not living with the care recipient	29 (37%)	13 (39%)
Hours of caring per week (h)		
0–2	3 (4%)	1 (3%)
3–10	16 (20%)	7 (21%)
11–20	8 (10%)	3 (9%)
21–40	5 (6%)	2 (6%)
41–81	11 (14%)	6 (18%)
≥81	36 (46%)	14 (42%)
Current psychotropic medication		
Yes	19 (24%)	9 (27%)
No	60 (76%)	24 (73%)
Previous psychotherapy		
None	47 (57%)	14 (41%)
CBT	6 (7%)	3 (9%)
Counselling	23 (28%)	14 (41%)
Other	6 (7%)	3 (9%)
GAD7 (0–21)	6.54 (5.35)	6.36 (2.33)
None (0–4)	33 (42%)	6 (18%)
Mild (5–9)	31 (39%)	25 (76%)
Moderate (10–14)	5 (6%)	2 (6%)
Severe (15+)	10 (13%)	0
PHQ9 (0–27)	7.62 (5.88)	7.79 (3.43)
None (0–4)	27 (34%)	5 (15%)
Mild (5–9)	25 (32%)	15 (45%)
Moderate (10–14)	7 (22%)	13 (39%)
Severe (15+)	10 (13%)	0
CESD-R (0–60)	15.43 (12.72)	15.21 (7.26)
AAQ-II (7–49)	20.40 (9.79)	20.52 (7.60)
CFQ (7–49)	24.26 (9.90)	25.09 (7.58)
EACQ (15–75)	41.42 (8.95)	41.39 (8.95)
Patient variable (range of the scale)	Mean (SD) or *N* (%) Screened (*N* = 79)^a^	Mean (SD) or *N* (%) Invited to the intervention (*N* = 33)
Dementia diagnosis		
Alzheimer’s disease	31 (39%)	13 (39%)
Vascular dementia	9 (11%)	4 (12%)
Dementia with Lewy bodies	2 (3%)	2 (6%)
Frontotemporal dementia	2 (3%)	1 (3%)
Mixed dementia	23 (29%)	10 (30%)
Other	12 (15%)	3 (9%)
Mean age (years)	78.78 (8.99)	82.06 (8.61)
Sex		
Female	44 (56%)	16 (48%)
Male	35 (44%)	17 (52%)
Frequency of memory and behaviour problems (RMBPC)		
Total (0–96)	40.82 (16.91)	41.45 (14.39)
Memory-related problems (0–28)	20.90 (6.94)	21.27 (6.92)
Affective distress (0–36)	11.79 (8.13)	11.12 (7.54)
Disruptive behaviours (0–32)	8.13 (6.51)	9.06 (5.72)

AAQ-II, Acceptance and Action Questionnaire-II; CBT, Cognitive Behaviour Therapy; CESD-R, Center for Epidemiologic Studies Depression Scale-Revised; CFQ, Cognitive Fusion Questionnaire; GAD7, General Anxiety Disorder-7; EACQ, Experiential Avoidance in Caregiving Questionnaire; PHQ9, Patient Health Questionnaire-9; RMBPC, Revised Memory and Behavior Problem Checklist.

^a^Due to missing data, 78 datasets were available for AAQ, CFQ and RMBPC and 77 datasets were available for EACQ.

Each online session had three sections: self-learning, reflection and home practice. In the self-learning section, interactive exercises to illustrate ACT skills were presented using multiple modes: video, audio and text. The reflection section encouraged participants to reflect on what was helpful in the session and leave questions for the online therapist if anything was unclear. Therapists provided individually tailored feedback to normalise difficult thoughts and emotions participants were experiencing and encourage them to practice ACT skills they found helpful. Therapists were directed to provide text-based feedback using the therapist portal of the online programme within 3 days, where possible, but no later than 7 days (i.e. before the next session was made available). In the home practice section, participants were encouraged to identify a small step they could take that still reflected their value and practise ACT skills offline between online sessions.

#### Optional peer support group

Participants were given the option to join three-peer support group sessions via video call alongside the online programme. Following the screening session, eligible participants were asked if they wished to sign up for the peer support group option. If three or more participants signed up for this option within 3 weeks from the date of the screening session, then peer support groups were organised by one of the therapists. If not, participants were asked to start the online programme without this option, and they were informed that they would be invited if the option became available before they completed the online programme. The aim of peer support groups was to meet other participants and encourage each other in completing the online programme. A trial therapist facilitated group sessions, but there were no specific, planned, therapeutic elements to peer support groups and sessions were participant-led.

#### Therapists

Therapists did not hold any formal qualification in Clinical Psychology or CBT (e.g. assistant psychologist, social prescribing link worker). Three of nine therapists had previously attended training sessions on ACT (e.g. 2-day workshop). All therapists attended a 2-day training on ACT and online feedback provision for the current study. They were also invited to attend monthly drop-in group supervision sessions led by the Chief Investigator each month via video call during the trial. One allocated participant was randomly selected for each therapist, and the online feedback provided to them by their therapist across eight online sessions was reviewed by two independent ACT experts. The same two raters completed a checklist designed for this study to evaluate intervention fidelity for each therapist. The total scores of three sub-categories of the checklist (i.e. feedback consistent with/inconsistent with ACT principles, general therapist competence) were averaged between raters to produce an overall score for each category.

### Procedure

Participants were asked to provide written consent via post or electronically before attending the screening session. Due to the COVID-19 pandemic, all the screening sessions were conducted remotely via video call or telephone by the assessor (research assistant independent from the intervention delivery). Participants were given the opportunity to complete questionnaires via post or an online survey platform. Participants who met the eligibility criteria were immediately invited to access the online intervention.

At the end of the intervention phase, participants were invited to complete the follow-up questionnaires via post or an online survey platform. All participants were also invited to attend an individual, telephone, semi-structured interview conducted by the assessor to share their experiences and provide feedback on online ACT. All therapists were also invited to an individual, semi-structured interview to provide their views on implementation. Qualitative findings on acceptability of the intervention and implementation will be reported elsewhere.

### Feasibility and acceptability outcomes

#### Primary outcomes

The following two pre-defined indicators of success needed to be met in order for progression to a full-scale effectiveness trial to be deemed feasible: successful uptake (recruitment of 30 participants over 6 months) and initial engagement (≥70% completing at least two online sessions). These indicators were developed based on the consensus between experienced trialists in the research team and were agreed with the funder before the conduct of the feasibility trial.

#### Secondary outcomes

The following data were also examined to inform the design of a full-scale effectiveness trial.

*Recruitment, eligibility and attrition*. The number of referrals, reasons for refusal, numbers ineligible, reasons for ineligibility and numbers lost to follow-up.

*Resulting sample characteristics*. Descriptive demographic data for all participants who attended the screening session and those eligible invited to the intervention phase.

*Treatment completion and acceptability*. The number of sessions completed in the 12-week intervention phase, reasons for withdrawal from the intervention and the uptake rate of the peer support group option.

*Therapist feasibility and acceptability*. The time gap between the participant leaving a comment and their therapist providing a response, the length of time required for therapists to write each response and intervention fidelity ratings.

### Clinical outcomes

The clinical outcome measures were included at both screening and follow-up to examine the feasibility of clinical outcomes data collection. Follow-up data were collected immediately post-intervention for completers and at the end of the intervention phase for those who were not able to complete all eight sessions in 12 weeks. The secondary clinical outcome measures were: Generalised Anxiety Disorder Scale (GAD7; Spitzer et al., 2006), Patient Health Questionnaire (PHQ9; Kroenke et al., 2001), Revised Centre for Epidemiologic Studies Depression Scale (CESD-R; Eaton et al., 2004), Acceptance and Action Questionnaire-II (AAQ-II; Bond et al., 2011), Cognitive Fusion Questionnaire (CFQ; Gillanders et al., 2014) and Experiential Avoidance in Caregiving Questionnaire (EACQ; Losada et al., 2014). The frequency of problematic behaviours subscale of the Revised Memory and Behavior Problems Checklist (RMBPC; Teri et al., 1992) was also used at the screening to characterise the care recipient. The detailed descriptions of each scale and data on its psychometric properties are provided in Table 2 of Supplemental Material.

**Table 2.** Means and standard deviations for clinical outcomes at follow-up and effect sizes.

	All sample (*N* = 24)		Completers sample (*N* = 18)	
	Mean (SD)	ES	Mean (SD)	ES
GAD7 (0–21)	4.96 (3.56)	0.51	4.50 (3.84)	0.70
PHQ9 (0–27)	6.79 (3.15)	0.12	6.56 (3.28)	0.15
CESD-R (0–60)	14.08 (9.10)	−0.02	12.72 (8.68)	0.19
AAQ-II (7–49)	20.83 (7.82)	−0.12	19.72 (8.01)	0.07
CFQ (7–49)	23.42 (8.08)	0.19	22.00 (8.30)	0.32
EACQ (15–75)	38.04 (6.88)	0.62	37.06 (7.41)	0.63

AAQ-II, Acceptance and Action Questionnaire-II; CESD-R, Center for Epidemiologic Studies Depression Scale-Revised; CFQ, Cognitive Fusion Questionnaire; GAD7, General Anxiety Disorder-7; EACQ, Experiential Avoidance in Caregiving Questionnaire; PHQ9, Patient Health Questionnaire-9.

### Sample size

A sample size of 30 is consistent with recommendations for sample sizes of 24–35 participants for pilot and feasibility studies when estimating the standard deviation for a continuous outcome (Julious, 2005; Teare et al., 2014; Whitehead et al., 2016).

### Statistical methods

The aim of a feasibility study is not to assess the efficacy of the intervention and thus no formal analysis was conducted, as recommended for pilot or feasibility studies (Lancaster et al., 2004; Thabane et al., 2010). Categorical measures were summarised using frequencies and percentages. Continuous measures were summarised using means and standard deviations (SD). However, to look for signals of effectiveness, Reliable Change Index (RCI) examined whether the observed change in key secondary clinical outcomes (i.e. GAD7 and PHQ9 used for screening) at the individual level were greater than could be explained by errors of measurement. RCIs were thus calculated for each participant by dividing the difference of the scores before and after the intervention by the standard error of measurement (Jacobson & Truax, 1991); RCI values greater than ± 1.96 indicate reliable improvement or reliable deterioration. The standard error of measurement for each scale was computed using SD and Cronbach’s alpha reported in the studies providing norms for primary care patients (Kroenke et al., 2001; Spitzer et al., 2006). The effect sizes (Cohen’s *d*) were also calculated for each clinical outcome using cases for which both pre-and post-data were available. These were calculated by subtracting the pre-group mean from the post-group mean and divided by the SD at pre (Durlak, 2009).

## Results

### Primary outcomes

Thirty-three eligible participants (110% of the target sample) were recruited over 6 months and 30 participants (91%) completed two or more online sessions. Thus, pre-defined indicators of success were met.

### Secondary outcomes

#### Recruitment, eligibility and attrition

**Figure 1.**
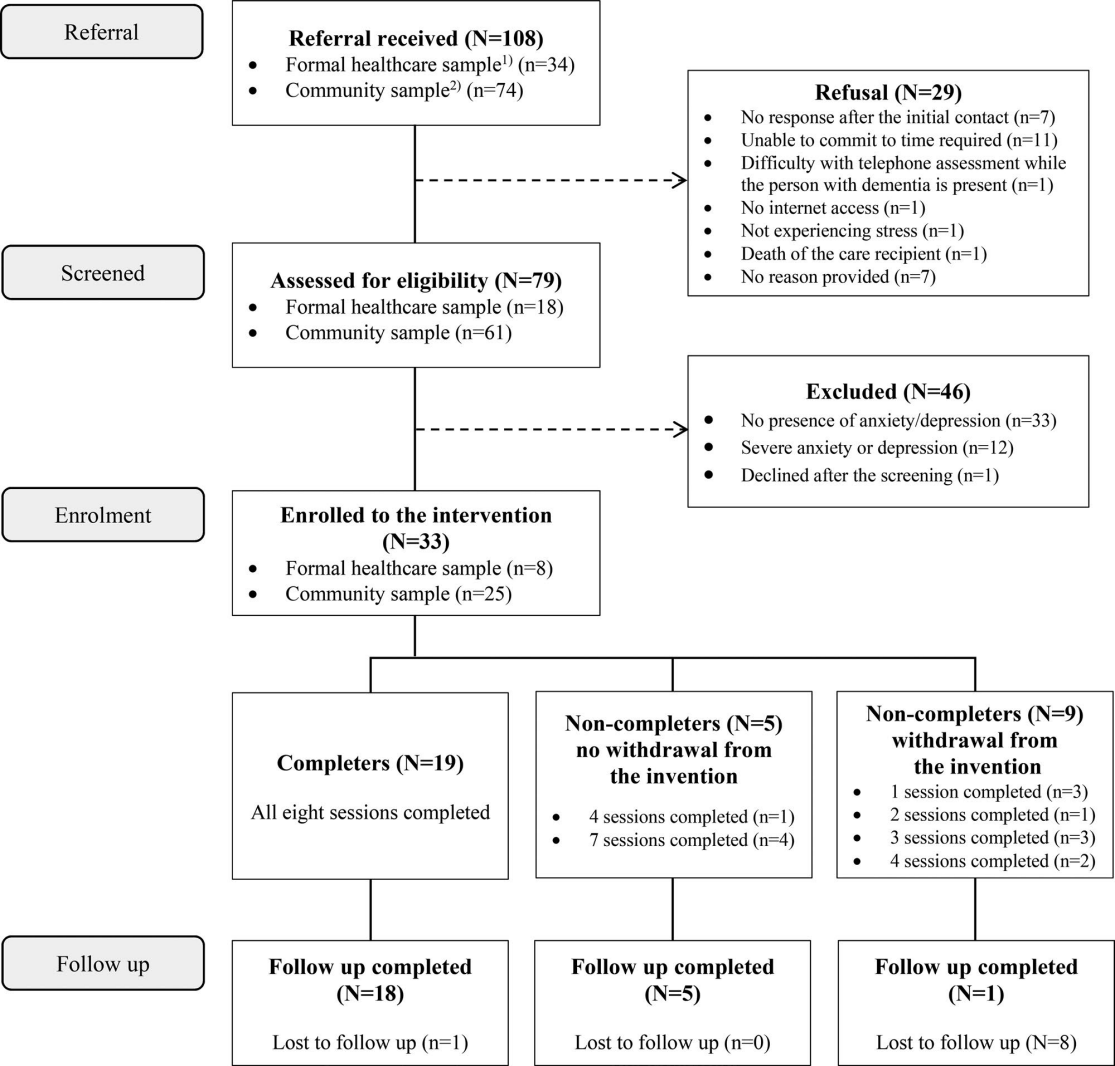
iACT4CARERS feasibility trial flow diagram. Note. ^1)^ Formal healthcare sample includes participants recruited via clinician referral from and primary (GP practice) and secondary (NHS mental health trusts) healthcare services; ^2)^ Community sample includes participants self-referred through public advertisement, Join Dementia Research and other ethically approved dementia studies.

The number of referrals, reasons for refusal, numbers ineligible, reasons for ineligibility and numbers lost to follow-up are presented in Figure 1. Of those 108 participants referred, 79 (73%) were screened and 33 out of those 79 screened (42%) were eligible. The final 33 participants enrolled in the intervention consisted of 25 participants (76%) who self-referred from the community and eight participants (24%) who were referred from primary and secondary care services. Nine of the 33 participants (27%) were lost to follow-up.

#### Resulting sample characteristics

Characteristics of participants are summarised in Table 1. As per Table 1, missing data were minimal, only five outcome data (out of a total of 474 outcome data at the screening) were missing across all clinical outcome measures. The majority of participants were female with at least secondary education completed. More than half of the participants were in their 60s–70s and retired, lived with the person with dementia, provided care for more than 41 h per week and had not received any form of psychological therapy in the past. Of the 33 participants enrolled in the intervention, 22 had both mild-to-moderate anxiety and depression. The remaining participants only had one condition (anxiety alone *n* = 5; depression alone *n* = 6).

#### Participant feasibility and acceptability

Of the 33 participants, 29 participants (70%) completed more than 7 sessions and 19 (58%) completed all 8 sessions. The average number of sessions completed during the 12-week intervention phase was 6.24 (SD = 2.55). Ten participants (30%) signed up for the peer support group option. Of these signed up, six attended all three-peer support group sessions and two did not attend any.

Nine participants (27%) withdrew from the intervention before the end of the 12-week intervention phase (Figure 1). The reasons for early withdrawal were: decline in the person with dementia’s physical health (*n* = 1), decline in own physical health (*n* = 2), changes in caring responsibilities due to COVID-19 (*n* = 3), death of the care recipient (*n* = 1) and death of a close family member (*n* = 1). One person did not provide a reason.

#### Therapist feasibility and acceptability

In total, 206 online sessions were completed by 33 participants. The time gap between the participant leaving a comment and their therapist providing a response for each session ranged from 0 to 24 days, with 96% of responses provided within 7 days. Significant delays mainly occurred during the Christmas holiday period. On average, therapists provided online feedback for each session within 2.72 days (SD = 3.21). The length of time required for therapists to write each response ranged from 5 to 120 min, with 93% of responses written in less than 30 min. On average, therapists spent 20.08 min (SD = 16.61) to write a response.

The results of treatment fidelity ratings are provided in Table 3 of Supplemental Material. There was no variance in the scores of ACT-inconsistent feedback and general therapist competency categories, suggesting the use of ACT-inconsistent feedback was none or minimal and all therapists were generally competent in providing supportive, well-structured feedback tailored to individuals. The scores of the ACT-consistent feedback category ranged from 13 to 20 across nine therapists (*M* = 16.83; SD = 2.17; possible scale range = 4–20; high score means higher fidelity).

**Table 3.** Reliable change in participants from baseline to follow-up including non-completers.

Symptom severity at baseline	Reliable deterioration *N*	No reliable change *N*	Reliable improvement *N*
Mild anxiety GAD7: 5–9 (*N* = 19)	0	12	7
Moderate anxiety GAD7: 10–14 (*N* = 1)	0	0	1
Mild depression PHQ9: 5–9 (*N* = 11)	2	8	1
Moderate depression PHQ9: 10–14 (*N* = 8)	0	4	4

### Clinical outcomes

Table 2 presents means and standard deviations for all clinical outcomes at follow-up and pre-post effect sizes. Table 3 presents the number of participants who demonstrated reliable improvement or deterioration in anxiety or depressive symptoms at follow-up. Of those 20 participants who presented mild or moderate anxiety at screening, 8 (40%) showed a reliable improvement in scores on the GAD7 at follow-up. Of those 19 participants who presented mild or moderate depression at screening, 5 (26%) demonstrated a reliable improvement in scores on the PHQ9 at follow-up. A small number of participants (*n* = 2) showed a reliable deterioration on the PHQ9.

## Discussion

The findings demonstrated that it is feasible to recruit participants and deliver internet-delivered self-help ACT, guided by novice therapists, within primary and secondary care services in the UK and the intervention appears acceptable to family carers of people with dementia. The pre-defined indicators of success in terms of uptake (recruitment of 30 participants over 6 months) and initial engagement (≥70% completing at least two online sessions) were successfully met.

Secondary outcomes further demonstrated that 108 referrals were received across three-study sites in just 6 months, 70% of participants completed seven or all sessions and none of the reasons for early withdrawal was related to the intervention. These figures further support that the proposed intervention was acceptable to family carers of people with dementia who were mainly in their 60s and 70s and were older than participants mainly targeted in previous self-help ACT studies (e.g. student populations) (Brown et al., 2016).

All study procedures were conducted completely remotely due to the COVID-19 pandemic, however missing data were minimal. The 27% attrition rate was comparable to those reported in previous studies on in-person ACT for family carers of people with dementia (Losada et al., 2015; Marquez-Gonzalez et al., 2020). The current study provides valuable evidence supporting the feasibility of remote delivery of both an ACT intervention and a trial among this population. Importantly, this mode of delivery enabled us to reach out to carers isolated in the community. In this study, the majority of eligible participants (76%) had self-referred from the community, as opposed to 24% of participants who were referred from primary and secondary care services. This highlights that currently there is a great unmet need in the community, and a large proportion of carers do not have access to formal healthcare services to meet this support need. This may also suggest that family carers are more sensitive than healthcare professionals in identifying the need for support. Recent literature on carer support needs also recommends moving away from traditional, informal, professionally led needs assessments and considering the use of carer-led assessment tools that reflect the person-centred approach (Ewing et al., 2015, 2016).

Although the study was not powered to examine effectiveness, there was preliminary evidence of improvements in scores of anxiety and depression. These improvements were more evident for anxiety. This is clinically important, as the current evidence suggests that the efficacy of conventional CBT on anxiety is limited for family carers of people with dementia (Hopkinson et al., 2019) and older people (Kishita & Laidlaw, 2017). There is also emerging evidence that in-person ACT can improve late-life treatment-resistant generalised anxiety disorder (Gould et al., 2021), and thus further investigation of the efficacy of internet-delivered guided self-help ACT on carer anxiety in a full-scale trial is warranted. Furthermore, considering the proportion of participants who did not present any symptoms at screening, future research should examine whether this intervention is beneficial in not only treating but also preventing anxiety and depressive symptoms in family carers of people with dementia.

Two participants demonstrated deterioration in depression. These participants had scored 6 and 7 on the PHQ9 at baseline but these increased to 10 and 12 at follow-up. Participant recruitment started after the first COVID-19 national lockdown ended, and the trial was completed during the second national lockdown; the impact of these external factors on deterioration cannot be ruled out as a control group was not included in the study. An examination of differential effects of internet-delivered guided self-help ACT on anxiety and depression including its long-term effects is required in a full-scale trial.

Findings also reveal that the workload burden on therapists was not excessive. Novice therapists who were not formally trained in clinical psychology or CBT but attended a 2-day training for the current study were able to provide immediate feedback to their participants, in less than 3 days on average, which adhered to ACT principles and required an average of 20 min of their time to write. The current average cost per recovered patient is £1043 for a low-intensity psychological treatment (e.g. guided self-help CBT, group CBT) in the East of England, and this increases to £2895 per recovered patient if a high-intensity psychological treatment is offered (e.g. 16 sessions of in-person CBT) (Radhakrishnan et al., 2013). An evaluation of the cost-effectiveness of internet-delivered guided self-help ACT in a full-scale trial is critical to further investigate whether this intervention can save the cost of treatment while maintaining effective outcomes. Following further efficacy testing, and assuming this is successful, there will need to be a plan and collaboration, possibly including implementation research, into the best way to roll out this technology in a cost-effective and sustainable way.

The uptake and attendance rates at the optional online peer support sessions were relatively low. Qualitative feedback from participants highlighted that feelings of uncertainty about other group attendees and inflexible dates and times due to the availability of therapists became barriers for them to sign up for groups. Although previous studies have demonstrated that peer support groups can be delivered either online or face-to-face and may have potential benefit to carers (Carter et al., 2020), this part of the programme may need to be reconsidered in the future trial.

There were some methodological limitations. Participant recruitment took place in three countries in the East of England. The population of the county that had the largest number of recruits is more than 90% White British, thus, the ethnic diversity of the sample was limited. This was a single-arm feasibility study, and preliminary evidence of improvements in outcomes (which was not the aim of the study) is not conclusive and should be interpreted with caution. The single-arm design also might overestimate recruitment and retention in an RCT. Due to the lack of follow-up beyond post-intervention, whether treatment gains are maintained following completion is unknown. Finally, due to the nature of the role of therapists (i.e. providing brief online feedback and not delivering ACT itself), it was not possible to use a standardised ACT fidelity measure. Therefore, a non-validated checklist was used.

## Conclusion

This study provided evidence for the feasibility and acceptability of internet-delivered guided self-help ACT with family carers of people with dementia and the methods of testing it. Although there were some signs of improvement in anxiety and depression, this was a single-arm underpowered feasibility study and thus this should be interpreted with caution. A full-scale trial with a diverse sample of carers to assess the clinical- and cost-effectiveness of the intervention including its long-term effects is warranted.

## Supplementary Material

Supplemental Material

Supplemental Material

Supplemental Material
