# Supplementary material for: Internet-delivered guided self-help acceptance and commitment therapy for family carers of people with dementia (iACT4CARERS): a feasibility study
Source: Aging Ment Health. 2021 Oct 7;26(10):1933–41. doi: 10.1080/13607863.2021.1985966 (PMC9629048; doi:10.1080/13607863.2021.1985966)
Supplement: Supplemental Material [file CAMH_A_1985966_SM8537.docx]

Supplemental material Table 3

Intervention fidelity checklist scores for each therapist

| Therapists  ID | Feedback  consistent with  ACT principles^1)^  (possible range: 4-20) | Feedback  inconsistent with  ACT principles^2)^  (possible range: 4-20) | General therapist competence^3)^  (possible range: 3-15) |
| --- | --- | --- | --- |
| Th.1 | 13 | 5 | 14 |
| Th.2 | 15 | 4 | 15 |
| Th.3 | 17 | 5 | 14 |
| Th.4 | 17 | 5 | 15 |
| Th.5 | 17 | 4 | 15 |
| Th.6 | 18 | 4 | 15 |
| Th.7 | 19 | 5 | 15 |
| Th.8 | 19 | 4 | 14 |
| Th.9 | 20 | 5 | 15 |

*Note*.

1) The first scale “feedback consistent with ACT principles” consisted of four items. An example of items included: The therapist encourages the participant to use techniques to unhook (step back) from difficult thoughts and feelings and/or to take actions towards values.

2) The second scale “feedback inconsistent with ACT principles” consisted of four items. An example of items included: The therapist encourages the participant to use techniques to control (get rid of) internal experiences.

3) The third scale “general therapist competence” consisted of three items. An example of items included: The therapist responds in a respectful, caring and non-judgmental manner.

Each item was rated on a five-point scale: 1=skill never demonstrated; 2=skill demonstrated at least once (across all sessions completed); 3=skill demonstrated several times (across all sessions completed); 4=skill demonstrated with relatively high frequency (across all sessions completed); 5=skill demonstrated with great frequency (across all sessions completed).
