# Supplementary material for: Internet-delivered guided self-help acceptance and commitment therapy for family carers of people with dementia (iACT4CARERS): a feasibility study
Source: Aging Ment Health. 2021 Oct 7;26(10):1933–41. doi: 10.1080/13607863.2021.1985966 (PMC9629048; doi:10.1080/13607863.2021.1985966)
Supplement: Supplemental Material [file CAMH_A_1985966_SM7988.docx]

Supplemental material Table 2

Secondary clinical outcomes included in the study

| Scale | Descriptions of the scale | Psychometric properties  (Cronbach’s alpha) |
| --- | --- | --- |
| GAD7  (Spitzer et al., 2006) | This scale assesses the severity of anxiety symptoms. It is routinely used an outcome measure in the NHS psychological services. | .92  (Spitzer et al., 2006) |
| PHQ9  (Kroenke et al., 2001) | This scale assesses the intensity of depressive symptoms. It is routinely used an outcome measure in the NHS psychological services. | .89  (Kroenke et al., 2001) |
| CESD-R  (Eaton et al., 2004) | This scale assesses symptoms defined by DSM-V for a major depressive episode. It is a frequently used measure for assessing depressive symptoms in intervention studies with dementia carers. | .92  (Van Dam & Earleywine, 2011) |
| AAQ-II  (Bond et al., 2011) | This scale assesses psychological inflexibility, the degree of dominance of psychological reactions (e.g. evaluative and self-descriptive thoughts, painful emotions) over chosen values and contingencies in guiding action. | .88  (Bond et al., 2011). |
| CFQ  (Gillanders et al., 2014) | This scale assesses levels of fusion with cognition, the degree of entanglement, struggle and effort to control uncomfortable thoughts. | .88-.93  (Gillanders et al., 2014). |
| EACQ  (Losada et al., 2014) | This scale assesses levels of experiential avoidance in the caregiving context, the degree of effort to control and avoid uncomfortable thoughts and emotions related to caregiving. | .70  (Losada et al., 2014). |
| RMBPC  (Teri et al., 1992) | This scale is a carer-report measure designed to assess the frequency of and reaction to problematic behaviours in people with dementia and carer reactions to these behaviours. | .84  (Teri et al., 1992). |

Note. AAQ-II = Acceptance and Action Questionnaire-II; CESD-R = Center for Epidemiologic Studies Depression Scale-Revised; CFQ = Cognitive Fusion Questionnaire; GAD7 = General Anxiety Disorder-7; EACQ = Experiential Avoidance in Caregiving Questionnaire; PHQ9 = Patient Health Questionnaire-9.
