# Supplementary material for: Internet-delivered guided self-help acceptance and commitment therapy for family carers of people with dementia (iACT4CARERS): a feasibility study
Source: Aging Ment Health. 2021 Oct 7;26(10):1933–41. doi: 10.1080/13607863.2021.1985966 (PMC9629048; doi:10.1080/13607863.2021.1985966)
Supplement: Supplemental Material [file CAMH_A_1985966_SM7170.docx]

**Supplemental material Table 1**

**iACT4CARERS intervention outline v2.0**

**Course Goal**

**By the end of this course participants will be prepared to:**

- Handle difficult thoughts and feelings more effectively, so they have less impact and influence over the person.
- Clarify their ‘values’ (what matters to them, what they want to stand for, how they want to treat themselves and others).
- Take actions to solve external problems and do things that make life better.

|  | **ACT process** | **Learning Objectives**  ***By the end of this session, participants will be able to:*** | **Learning Activities**  **What will participants do online (read, watch, listen to, and perform)?** | **Learning Activities**  **What will participants do between sessions (practice)?** |
| --- | --- | --- | --- | --- |
| S1 | Introduction to ACT  Creative hopelessness  Openness | - Describe the ACT choice point model and the main goal of this therapy - See the potential adverse impact of common control strategies (denial, avoidance and suppression of feelings) - Engage with the present moment awareness exercise | - Complete a brief rating form - Watch a video clip ‘ACT choice point model’ - Watch a video clip ‘common feelings experienced by carers of people with dementia’ - Complete a short questionnaire on common thoughts and feelings - Watch a video clip ‘adverse impact of suppression of feelings (pushing beach ball in a pool)’ - Listen to an audio file ‘connect, breath, and open up exercise’ | - Listen to the audio file ‘connect, breath, and open up exercise’ once every day |
| S2 | Values  Introduction to committed actions | - See the differences between values and goals - Identify the important areas of their life - Be aware of a lack engagement with some areas of their life (e.g. self-care) - Identify the smallest step they are willing to take that would move them towards valued directions | - Complete a brief rating form - Listen to ‘connect, breath, and open up exercise’ - Read text on ‘recap of session 1’ - Watch a video clip ‘differences between values and goals’ - Watch a video clip ‘garden metaphor’ - Complete a short questionnaire on the important areas of their life and important qualities they wish to reflect in what they do | - Identify and engage with the smallest step participants would be willing to take that would move them towards valued directions over the next week |
| S3 | Overcoming external barriers (SOC)  Committed actions | - See the differences between common external barriers and internal barriers experienced by carers of people with dementia - Describe when strategies to address external barriers may be useful - Come up with some ideas to overcome their own external barriers using selective optimisation and compensation (SOC) in order to take committed actions - Identify the smallest step they are willing to take that would move them towards valued directions | - Complete a brief rating form - Listen to ‘connect, breath, and open up exercise’ - Read text on ‘recap of session 2’ - Watch a video clip ‘differences between common external and internal barriers experienced by carers of people with dementia’ - Read text on ‘decision tree – when to use strategies to address internal/external barriers’ - Watch a video clip ‘three-step problem solving approach’ - Watch a video clip ‘three-step problem solving approach – Mrs Stewart’s example (an example of a spousal carer of a family member with dementia)’ - Complete the SOC worksheet | - Identify and engage with the smallest step participants would be willing to take that would move them towards valued directions over the next week |
| S4 | Overcoming internal barriers (cognitive fusion)  Committed actions | - Describe when strategies to address internal barriers may be useful - See the potential adverse impact of common control strategies (controlling thoughts) - Engage with different defusion exercises - Identify the smallest step they are willing to take that would move them towards valued directions | - Complete a brief rating form - Listen to ‘connect, breath, and open up exercise’ - Read text on ‘recap of session 3’ - Watch a video clip ‘common thoughts experienced by carers of people with dementia’ e.g. thoughts related to guilt, shame and frustration - Watch a video clip ‘hands as thoughts’ - Listen to ‘labelling thought exercise’ | - Identify and engage with the smallest step participants would be willing to take that would move them towards valued directions over the next week - Encourage participants to use defusion exercises when appropriate |
| S5 | Overcoming internal barriers (awareness and openness)  Committed actions | - See what it means by present moment awareness - Engage with different types of present moment awareness exercises - Identify the smallest step they are willing to take that would move them towards valued directions | - Complete a brief rating form - Listen to ‘brief observer exercise’ - Read text on ‘recap of session 4’ - Watch a video clip ‘what is present moment awareness’ - Listen to ‘tracking thoughts in time exercise’ - Listen to ‘dropping anchor’ | - Identify and engage with the smallest step participants would be willing to take that would move them towards valued directions over the next week - Encourage participants to use present moment exercises when appropriate |
| S6 | Self-compassion  Committed actions | - See the potential adverse impact of repetitive self-critical judgements - Engage with a self-compassion exercise - Identify the smallest step they are willing to take that would move them towards valued directions | - Complete a brief rating form - Listen to ‘brief observer exercise’ - Read text on ‘recap of session 5’ - Watch a video clip ‘compassion as an alternative to rumination aimed at avoiding fear of being a bad self or motivating oneself to do better’ - Listen to ‘self-acceptance and compassion exercise’ | - Identify and engage with the smallest step participants would be willing to take to demonstrate compassion towards oneself over the next week |
| S7 | Building a pattern of effective action | - Reconnect with their values - Describe a pattern of effective action that reflect their values - Describe helpful steps to take when emotional distress is too painful - Identify the smallest step they are willing to take that would move them towards valued directions | - Complete a brief rating form - Listen to ‘self-acceptance and compassion exercise’ - Read text on ‘recap of session 6’ - Watch a video clip ‘revisiting your garden’ - Complete a short questionnaire on the important areas of their life and important qualities they wish to reflect in what they do - Watch a video clip ‘building a pattern of effective action’ - Read text on ‘Helpful steps to take when emotional distress is too painful’ | - Identify and engage with the smallest step participants would be willing to take that would move them towards valued directions over the next week |
| S8 | Summary of ACT  Preparing for the future | - Summarise ACT skills learned - Identify some future challenges and see how some of ACT skills may be useful to address them | - Complete a brief rating form - Listen to ‘self-acceptance and compassion exercise’ - Read text on ‘recap of session 7’ - Watch a video clip ‘revisiting choice point model’ - Watch a video clip on ‘decision tree and overview of ACT skills’ - Complete the identifying barriers and plans worksheet |  |

S = Session
